# TRPC3 inhibition induces myofibroblast differentiation in diabetic dermal fibroblasts

**DOI:** 10.3389/fphys.2025.1577118

**Published:** 2025-04-30

**Authors:** Gemma Toogood, Robin Evans, Liping Zhang, Rima Patel, Songmei Meng, Vijay K. Boda, Wei Li, Junwang Xu

**Affiliations:** ^1^ Department of Physiology, College of Medicine, University of Tennessee Health Science Center, Memphis, TN, United States; ^2^ Department of Surgery, College of Medicine, University of Tennessee Health Science Center, Memphis, TN, United States; ^3^ Department of Pharmaceutical Sciences, College of Pharmacy, University of Tennessee Health Science Center, Memphis, TN, United States

**Keywords:** diabetic dermal fibroblast, myofibroblast, TRPC3, pharmacological inhibition, TGF-β signaling

## Abstract

Diabetic wounds present a significant healthcare challenge due to impaired healing mechanisms, with dermal fibroblasts playing a crucial role in tissue repair. This study investigates the role of transient receptor potential canonical-3 (TRPC3) in the dysfunction of diabetic fibroblasts and explores the therapeutic potential of TRPC3 inhibition. Findings reveal that TRPC3 expression is significantly elevated in diabetic dermal fibroblasts, which correlates with suppressed transforming growth factor-beta (TGF-β) signaling and impaired differentiation into myofibroblasts. Inhibiting TRPC3 effectively restores fibroblast functionality by upregulating TGF-β1 and its downstream effector, SMAD4. This restoration enhances the expression of key myofibroblast markers, such as α-smooth muscle actin (ACTA2) and type I collagen (COL1a1), which are essential for wound contraction and extracellular matrix remodeling. These results establish TRPC3 as a critical regulator of fibroblast activity and present TRPC3 inhibition as a promising therapeutic strategy for improving wound healing in diabetic patients.

## Introduction

The skin, the largest organ in the body, serves as a protective barrier to vital internal structures, regulates temperature and proprioception, and importantly plays a crucial role in the immune, nervous, and endocrine systems (Dąbrowska et al., 2018). To maintain these vital functions, wound healing must occur in a timely and organized manner (Wilkinson and Hardman, 2020). When this process is disrupted, chronic wounds can develop, disproportionately affecting those with chronic diseases such as diabetes mellitus (Sen, 2021). In people with diabetes, chronic hyperglycemia contributes to the formation of non-healing wounds through mechanisms such as peripheral neuropathy, increased susceptibility to infection, impaired microcirculation, delayed re-epithelization, and prolonged inflammation (Burgess et al., 2021; Shakya et al., 2015; Tsourdi et al., 2013). These chronic wounds place a significant burden on healthcare systems worldwide (Ong et al., 2023), increasing hospitalization, medical costs (Nussbaum et al., 2018), and the risk of severe complications such as limb amputation (Gandhi et al., 2020), while profoundly diminishing patients’ quality of life (Olsson et al., 2019). Despite the incomplete understanding of the pathogenesis of diabetic wounds, it is crucial to investigate potential pathways implicated in these wounds in order to develop therapeutic targets that address the underlying causes.

Normal wound healing is a well-coordinated process involving distinct phases: hemostasis, inflammation, re-epithelialization, and remodeling. These phases are orchestrated by the dynamic interplay of extracellular matrix (ECM) components, growth factors, and cytokines (Rodrigues et al., 2019). However, in diabetic skin, this process is disrupted, particularly during the inflammatory phase, due to an imbalance in pro-inflammatory and anti-inflammatory cytokines (Silveira et al., 2024). This dysregulated inflammatory response, driven by oxidative stress, impairs the transition of fibroblasts to myofibroblasts, a critical step for effective wound healing (Silveira et al., 2024).

Additionally, chronic inflammation in diabetes mellitus, mediated by increased macrophage polarization, is also implicated in poor wound healing (Wu et al., 2022).

Myofibroblasts are key in normal wound repair as they stimulate wound contraction, ECM remodeling, and collagen deposition (Wan et al., 2021). Impaired myofibroblast transdifferentiation, therefore, results in poor wound healing (Cutolo et al., 2020; Darby et al., 2014). This transition is primarily mediated by transforming growth factor-beta (TGF-β) (Frangogiannis NG, 2020) which induces the expression of COL1a1, the most abundant collagen in a wound matrix (Liu et al., 2012). As well, TGF-β activates the transcription factors Smad2/3 and Smad4 which translocate to the nucleus and regulate the gene expression profile of the myofibroblast phenotype, including alpha-smooth muscle actin (αSMA) (Evans et al., 2003). αSMA integrates into actin stress fibers and enhances the contractile capability of myofibroblasts.

One potential regulator of TGF-β is the transcellular calcium channel transient receptor potential canonical-3 (TRPC3). Elevated TRPC3 expression has been associated with decreased expression of TGF-β (He et al., 2019). TRPC3 is thought to play a role in various human pathologies, including cancer, cardiac arrhythmias, and scar formation due to the mediation of inflammatory regulation and cellular proliferation (Li et al., 2023; Tiapko and Groschner, 2018). TRPC3-mediated inflammation occurs via Reactive Oxygen Species (ROS) and nuclear factor kappa beta (NF-kβ) (Yan et al., 2024). Prior studies have demonstrated that TRPC3 has higher expression in dermal fibroblasts following injury, via activation of NF-kβ and leading to maladaptive healing thought to be secondary to prolonged inflammation (Kitajima et al., 2016; Yan et al., 2024). Given TRPC3’s role in the regulation of TGF-β and inflammation, it is an interesting candidate to be further investigated in the role of fibroblast to myofibroblast transition in diabetic wounds.

We hypothesize that increased expression of TRPC3 in diabetic (Db) dermal fibroblasts results in inhibition of TGF-β thereby impairing the fibroblast to myofibroblast transition. This impaired transition may underlie the development of chronic wounds in diabetes mellitus. Understanding the role of TRPC3 in this process may lead to the identification of novel therapeutic targets for improved wound healing in diabetic patients.

## Results

### TRPC3 expression in Db dermal fibroblasts

**FIGURE 1:**
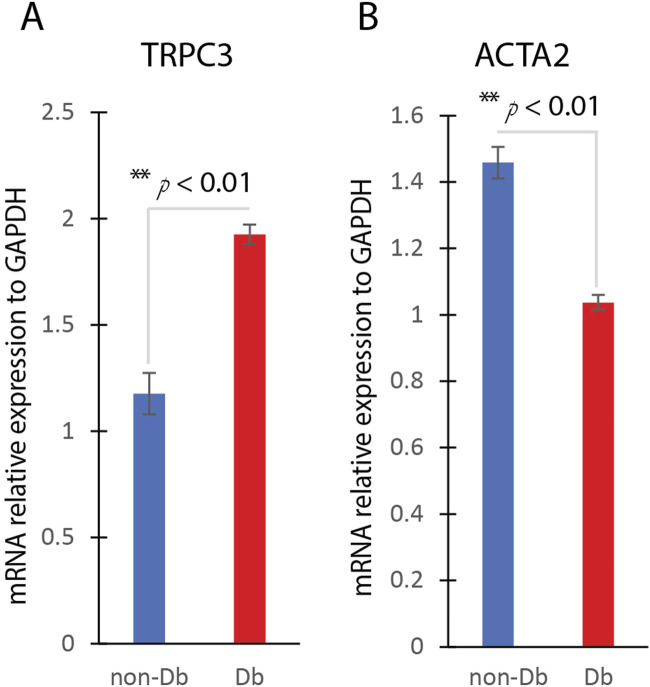
TRPC3 and ACTA2 Expression in Diabetic and Non-Diabetic Dermal Fibroblasts. **(A)** QRT-PCR analysis of TRPC3 gene expression in non-Db and Db dermal fibroblast (mean ± SD, n = 4 per group). **(B)** QRT-PCR analysis of ACTA2 gene expression in non-Db and Db dermal fibroblast (mean ± SD, n = 4 per group). **P < 0.01.

We first examined the expression of TRPC3 in Db dermal fibroblasts using quantitative real-time PCR (qRT-PCR) and found that TRPC3 mRNA levels were significantly upregulated compared to non-diabetic (non-Db) control fibroblasts (Figure 1A). This suggests that TRPC3 expression is elevated in the diabetic wound environment, potentially influencing fibroblast function.

To assess myofibroblast differentiation, we analyzed the expression of ACTA2 (αSMA), a well-established marker of myofibroblast differentiation, using qualitative Real-Time PCR (qRT-PCR). qRT-PCR results showed that ACTA2 mRNA levels were significantly lower in Db fibroblasts compared to non-Db controls (Figure 1B). This data supports the notion that Db fibroblasts exhibit impaired myofibroblast differentiation under basal conditions.

### TGF-β induces myofibroblast differentiation in Db dermal fibroblasts

**FIGURE 2:**
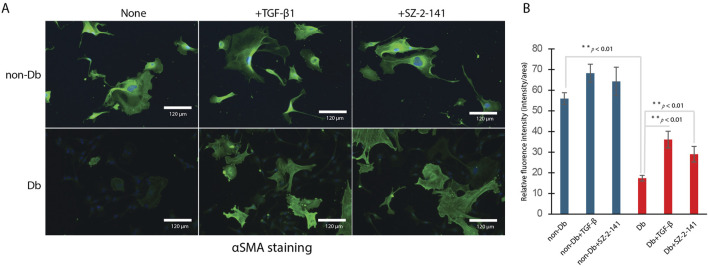
Immunohistochemical Staining with α-Smooth Muscle Actin (αSMA) in Dermal Fibroblasts. **(A)** Left: non-Db vs. Db fibroblast with treatment; Middle: non-Db vs. Db fibroblast treated with 10 ng/mL TGF-β1; Right: non-Db vs. Db fibroblast treated with 1 μM SZ-2-141. (n = 4 per group). **(B)** Flourence intensity measured and analyzed by ImageJ (n = 10 per group).

To further assess ACTA2 expression, immunostaining with anti-αSMA was performed to visualize protein localization in the cells. As shown in Figure 2A left, Db dermal fibroblasts exhibited lower fluorescence intensity compared to non-Db fibroblasts, indicating reduced αSMA expression. The fluorescence intensity of αSMA staining was quantified using ImageJ, revealing a significantly reduced signal in Db dermal fibroblasts compared to controls (Figure 2B, n = 10 cells).

**FIGURE 3:**
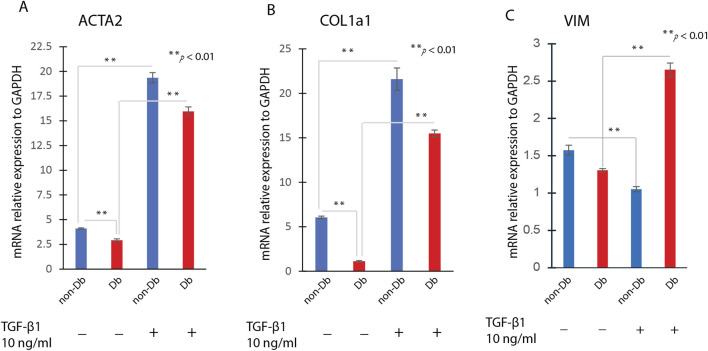
Effects of TGF-β1 Treatment on Fibroblast to Myofibroblast Transition. Dermal fibroblasts were treated with 10 ng/mL TGF-β1 for 24 h, then analysis for genes expression. The gene expression levels of ACTA2 **(A)**, COL1a1 **(B)** and VIM **(C)** were determined by qRT-PCR in TGF-β1treated fibroblast or non-treated fibroblasts (mean ± SD, n = 4 per group). **P < 0.01.

To determine whether Db fibroblasts retain the capacity for myofibroblast differentiation, both non-Db and Db fibroblasts were treated with TGF-β1. Following treatment, αSMA staining was detected in both groups, confirming their differentiation into myofibroblasts (Figure 2A middle). Treatment with TGF-β1 markedly increased αSMA fluorescence in Db fibroblasts (Figure 2B, n = 10 cells). In addition to αSMA staining, TGF-β1 treatment significantly increased the expression of key myofibroblast marker genes, including ACTA2 (Figure 3A), COL1a1 (Figure 3B), and VIM (vimentin, a cytoskeletal protein often associated with cells undergoing differentiation) (Figure 3C). These markers serve as critical indicators of myofibroblast differentiation, reinforcing that Db fibroblasts retain the potential to differentiate in response to TGF-β1 stimulation. Thus, despite their diabetic origin, Db fibroblasts remain responsive to external pro-differentiation signals, activating myofibroblast-specific pathways when exposed to TGF-β1.

### TRPC3 inhibition promotes myofibroblast differentiation

**FIGURE 4:**
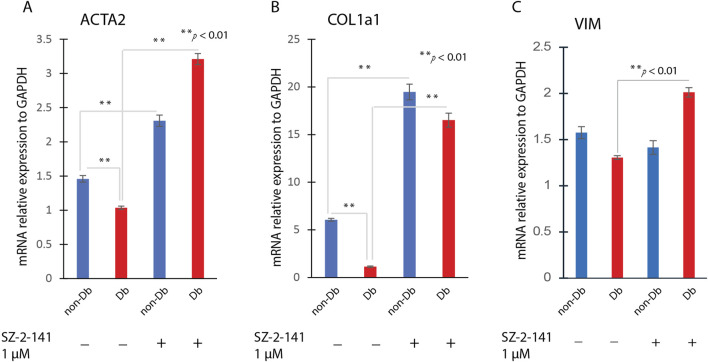
Effects of SZ-2-141 Treatment on Fibroblast to Myofibroblast Transition. Dermal fibroblasts were treated with 1 μM SZ-2-141 for 24 h, then analysis for genes expression. The gene expression level of ACTA2 **(A)**, COL1a1 **(B)** and VIM **(C)** were determined by qRT-PCR in SZ-2-141 treated fibroblast or non-treated fibroblasts (mean ± SD, n = 4 per group). **P < 0.01.

To test whether TRPC3 inhibition affects myofibroblast differentiation, we identified a suitable blocker, Pyr3, the most common TRPC3 inhibitor. Pyr3 has metabolic instability and off-target toxicity (Kiyonaka et al., 2009). We optimized the chemical structure of Pyr3, developing JW-65 and later SZ-2-141, a safer, metabolically stable TRPC3 inhibitor with high selectivity (Wang et al., 2025). Interestingly, when Db dermal fibroblasts were treated with a TRPC3 inhibitor, SZ-2-141, we observed a marked increase in αSMA staining, similar to the effects seen with TGF-β1 treatment (Figure 2A right) and confirmed by fluorescence intensity analysis (Figure 2B). Even more notably, TRPC3 inhibition by SZ-2-141 resulted in a significant upregulation of key myofibroblast marker genes, including ACTA2, COL1a1, and VIM (Figures 4A–C). These findings suggest that the inhibition of TRPC3 not only mimics the effects of TGF-β1 but also actively promotes myofibroblast differentiation in Db fibroblasts. Thus, in addition to external pro-differentiation signals like TGF-β1, blocking TRPC3 can serve as a potent driver of the myofibroblast phenotype in diabetic fibroblasts.

### TRPC3 inhibition upregulates TGF-β1 and SMAD4 expression

**FIGURE 5:**
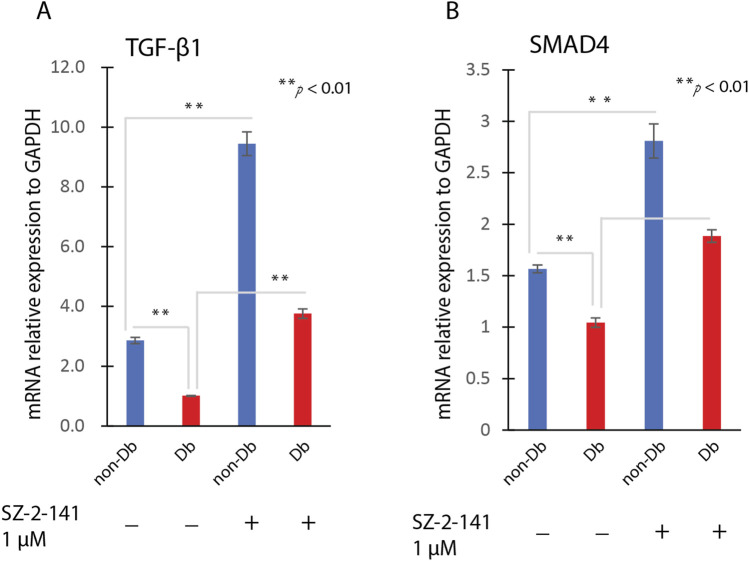
SZ-2-141 Treatment on TGF Signaling Pathway. Dermal fibroblasts were treated with 1 μM SZ-2-141 for 24 h, then analysis for genes expression. The gene expression level of TGF-β1 **(A)** and SMAD4 **(B)** were determined by qRT-PCR in SZ-2- 141 treated fibroblast or non-treated fibroblasts (mean ± SD, n = 4 per group). **P < 0.01.

**FIGURE 6:**
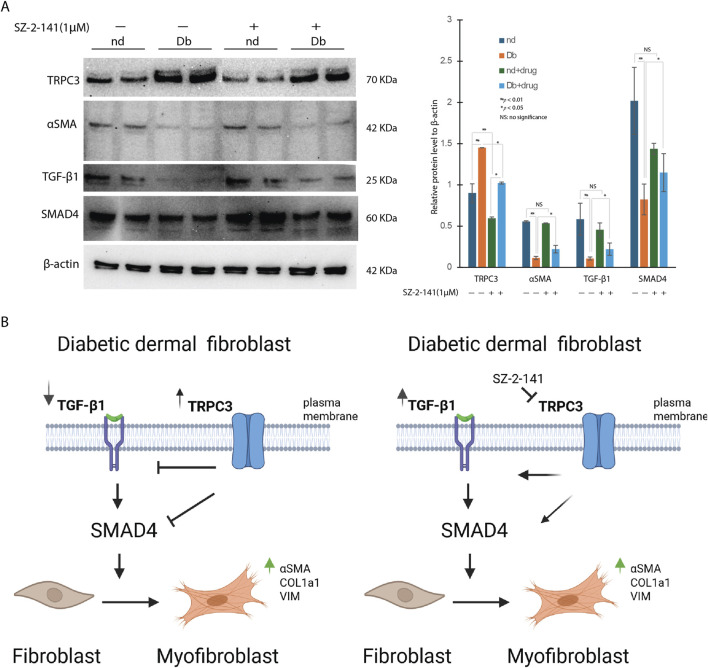
SZ-2-141 Treatment Modulates TGF Signaling Pathway at Protein Level in Dermal Fibroblast. **(A)** Western blot analysis revealed the differential expression of TRPC3, αSMA, TGF-β1 and SMAD4 in non-Db (nd), and diabetic (db) dermal fibroblast and the effects of SZ-2-141 treatment on their expression (mean ± SD, n = 4 per group, *P < 0.05, **P < 0.01). **(B)** Cartoon illustration of possible mechanisms of TRPC3/TGF-β signaling in diabetic dermal fibroblast.

To further explore the mechanism by which TRPC3 inhibition enhances myofibroblast differentiation, we examined the expression of key components of the TGF-β/Smad signaling pathway. Treatment of Db dermal fibroblasts with the TRPC3-selective inhibitor SZ-2-141 significantly increased the mRNA expression levels of TGF-β1 and SMAD4 (Figures 5A,B). At protein level, Western blot analysis revealed elevated TRPC3 protein expression and reduced αSMA levels in untreated Db fibroblasts (Figure 6A), aligning with the gene expression patterns shown in Figure 1. Notably, treatment with SZ-2-141 led to a marked decrease in TRPC3 protein levels and a concurrent increase in αSMA, TGF-β1, and SMAD4 protein expression in Db fibroblasts (Figure 6A). These results indicate that TRPC3 inhibition enhances the activation of the TGF-β/SMAD4 signaling pathway, which subsequently drives myofibroblast differentiation. Figure 6B provides a mechanistic illustration of these findings. The right panel demonstrates that in diabetic fibroblasts treated with the TRPC3 inhibitor SZ-2-141, there is an increase in TGF-β signaling, leading to enhanced myofibroblast differentiation. In contrast, the left panel depicts the diabetic condition, where TRPC3 is upregulated, potentially contributing to the suppression of TGF-β signaling and the impaired differentiation of myofibroblasts.

## Materials and methods

### Dermal fibroblast isolation and culture

Primary dermal fibroblasts were isolated from the skin of 17-week-old non-Db and diabetic (Db/Db) mice. They were cultured in full medium comprising Dulbecco’s modified eagle high-glucose (DMEM) (Sigma-Aldrich, St. Louis, MO, United States) supplemented with 10% fetal bovine serum (FBS; Gibco, MA, United States) and maintained at 37°C in a humidified atmosphere containing 5% CO_2_. For further experiments, cells were seeded in 6 - well plates or 8 -well chamber slide and cultured for 12 h. Thereafter, cells were starved for 16 h and were stimulated with 10 ng/mL TGF-β1 (PeproTech, Rocky Hill, NJ, United States) or 1 μM SZ-2-141, provided by Dr. Wei Li, TRPC3 selective inhibitor for 24 h.

## Immunocytochemistry

Db and non-Db dermal fibroblasts were cultured on 8-well chamber slide treated with or without TGF-β1 or SZ-2-141. Twenty-four hours later, cells were fixed with 4% paraformaldehyde for 15 min then permeabilized with 0.1% Triton X-100 in PBS. After washing with PBS, cells were preincubated in blocking solution (1% bovine serum albumin, BSA) in Phosphate-Buffered Saline with 0.05% Tween 20(PBST) for 1 h at room temperature) and incubated with primary mouse antibody against αSMA (ab5694, Abcam, Waltham, MA, United States) over-night. After rinsing with PBS, the cells were incubated with a second anti-mouse antibody labeled with Alexa 488 (Abcam) for 1 h. Nuclei were stained with 4,6-diamidino-2- phenolindole (DAPI) dihydrochloride. Fluorescence images were captured by an Olympus microscope (Westborough, MA, United States) and analyzed.

### Real-time quantitative PCR

TRIzol reagent (Invitrogen, Carlsbad, CA, United States) was used according to the manufacturer’s protocol to extract total RNA. RNA was converted to cDNA using SuperScript First-Strand Synthesis System (Invitrogen). GAPDH housekeeping gene was used to achieve internal normalization. Genes expression of TRPC3, ACTA2, COL1a1, TGF-β1, SMAD4, VIM were analyzed by qRT-PCR. Samples (*n* = 4 per group) were amplified in triplicate and averaged for each sample. DDCT method was used to calculate gene expression.

### Western blot

Non-Db and Db dermal fibroblasts, either untreated or treated with the TRPC3-selective inhibitor SZ-2-141, were lysed for protein extraction. Cell lysates were prepared in standard NP-40 lysis buffer (Abcam) supplemented with proteinase and phosphatase inhibitors. Protein lysates were quantified using the Pierce BCA protein assay kit (Thermo Fisher Scientific, Waltham, MA, United States). Equal masses of total protein were separated on 4%–12% SDS-polyacrylamide mini-gels, and blotted onto PVDF membranes (Millipore, Bedford, MA, United States). Membranes were subsequently blocked, incubated with primary antibodies, and incubated with secondary antibodies according to WesternBreeze Chromogenic Kit (Thermo Fisher Scientific). Alkaline phosphatase was detected on the PVDF membranes using a ready-to-use BCIP/NBT substrate (Thermo Fisher Scientific) for ready visualization of enzyme-linked antibodies. Rabbit anti-TRPC3, mouse anti- αSMA, rabbit anti-TGF-β1, anti-SMAD4, and anti- β-actin antibodies were obtained from Cell Signaling (Danvers, MA, United States). Quantification of relative intensities was achieved by ImageJ analysis (version 1.48v, National Institutes of Health, Bethesda, MD, United States).

### Statistical analysis

Differences in gene expression and protein levels between the groups were assessed by Student’s t-test. p < 0.05 was considered statistically significant.

## Discussion

The findings of this study highlight the critical role of TRPC3 in the dysfunction of diabetic dermal fibroblasts and its potential as a therapeutic target for improving wound healing in diabetic patients. The elevated expression of TRPC3 in diabetic fibroblasts and its correlation with impaired TGF-β1 signaling and myofibroblast differentiation provide new insights into the molecular mechanisms underlying diabetic wound healing deficiencies. TRPC3, a non-selective cation channel, has been implicated in various cellular processes, including calcium homeostasis and cell differentiation (Dietrich et al., 2010). This study demonstrates that TRPC3 is significantly upregulated in diabetic dermal fibroblasts, which aligns with previous findings suggesting that TRPC channels are involved in pathological conditions, including diabetes (Mita et al., 2010). The elevated TRPC3 levels in diabetic fibroblasts appear to disrupt TGF-β signaling, a key pathway for fibroblast-to-myofibroblast differentiation. TGF-β is essential for wound healing, as it promotes the expression of ECM components and facilitates wound contraction (Hinz et al., 2007). The suppression of TGF-β signaling in diabetic fibroblasts, mediated by TRPC3 overexpression, likely contributes to the delayed wound healing observed in diabetic patients (Brem and Tomic-Canic, 2007).

The inhibition of TRPC3 emerges as a promising therapeutic strategy, as it effectively restores TGF-β1 signaling and enhances fibroblast differentiation into myofibroblasts. The upregulation of SMAD4, a downstream effector of TGF-β1, following TRPC3 inhibition, underscores the importance of this pathway in fibroblast functionality as indicated in our study. The restoration of TGF-β signaling leads to increased expression of myofibroblast markers, such as ACTA2 and COL1 a 1, which are critical for wound contraction and ECM remodeling (Tomasek et al., 2002). These findings suggest that TRPC3 inhibition could reverse the impaired healing phenotype of diabetic fibroblasts, offering a potential avenue for therapeutic intervention.

Mechanistically, TRPC3 may influence intracellular calcium homeostasis, thereby affecting the activation of signaling cascades such as calcineurin/NFAT (Nuclear Factor of Activated T cells) or CaMK(calmodulin-dependent protein kinase) pathways (Pigozzi et al., 2006), which in turn modulate TGF-β1 transcription. Alternatively, TRPC3 activity may directly suppress transcription factors involved in TGF-β1 gene expression. Further studies are needed to dissect the precise molecular intermediates linking TRPC3 inhibition to TGF-β1 induction.

Diabetic wounds are characterized by chronic inflammation, reduced angiogenesis, and impaired fibroblast function, all of which contribute to delayed healing (Falanga, 2005). The identification of TRPC3 as a key regulator of fibroblast dysfunction provides a novel target for addressing these challenges. By restoring fibroblast activity and promoting myofibroblast differentiation, TRPC3 inhibition could enhance wound contraction and ECM deposition, thereby accelerating the healing process. This approach could complement existing therapies, such as growth factor supplementation and advanced wound dressings, to improve outcomes for diabetic patients (Brem et al., 2007).

While this study provides compelling evidence for the role of TRPC3 in diabetic fibroblast dysfunction, several questions remain. First, the precise mechanism by which TRPC3 suppresses TGF-β signaling requires further investigation. Additionally, the *in vivo* efficacy of TRPC3 inhibition in diabetic wound healing models needs to be validated. Future studies should explore the long-term effects of TRPC3 inhibition, potential off-target effects, and its interaction with other pathways involved in wound healing (Eming et al., 2014). Moreover, the development of specific and potent TRPC3 inhibitors will be crucial for translating these findings into clinical applications (Zhang et al., 2021).

## Conclusion

In conclusion, this study establishes TRPC3 as a critical regulator of fibroblast activity in diabetic wound healing and highlights its potential as a therapeutic target. By restoring TGF-β signaling and promoting myofibroblast differentiation, TRPC3 inhibition offers a promising strategy for improving wound healing in diabetic patients. These findings contribute to a deeper understanding of the molecular mechanisms underlying diabetic wound healing deficiencies and pave the way for the development of novel therapies to address this significant healthcare challenge.

## Data Availability

The raw data supporting the conclusions of this article will be made available by the authors, without undue reservation.
