# Supplementary figures and images for: TRPC3 inhibition induces myofibroblast differentiation in diabetic dermal fibroblasts
Source: Front Physiol. 2025 Apr 30;16:1577118. doi: 10.3389/fphys.2025.1577118 (PMC12075372; doi:10.3389/fphys.2025.1577118)

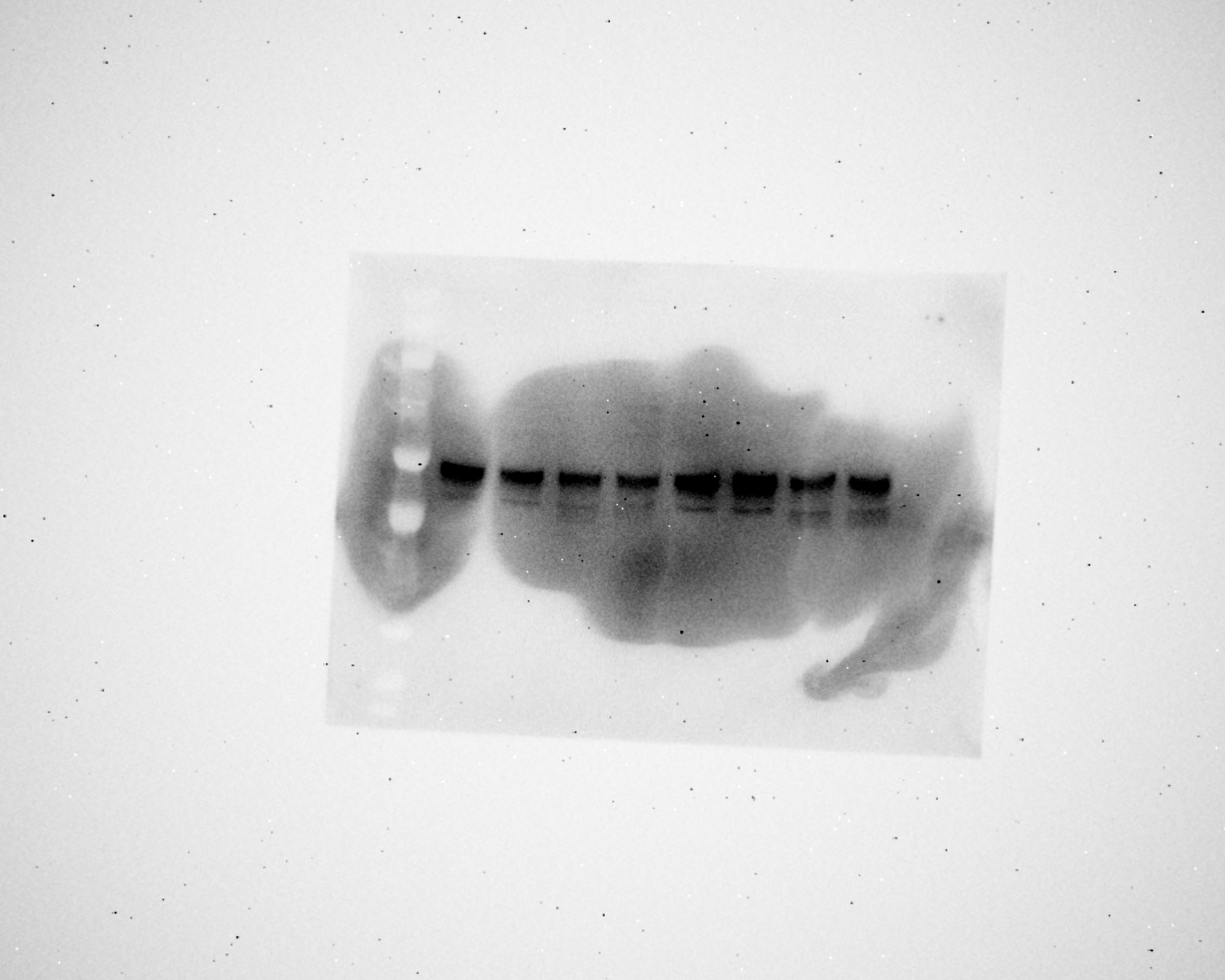

Supplement: Supplementary file 1 [file Image3.jpeg]

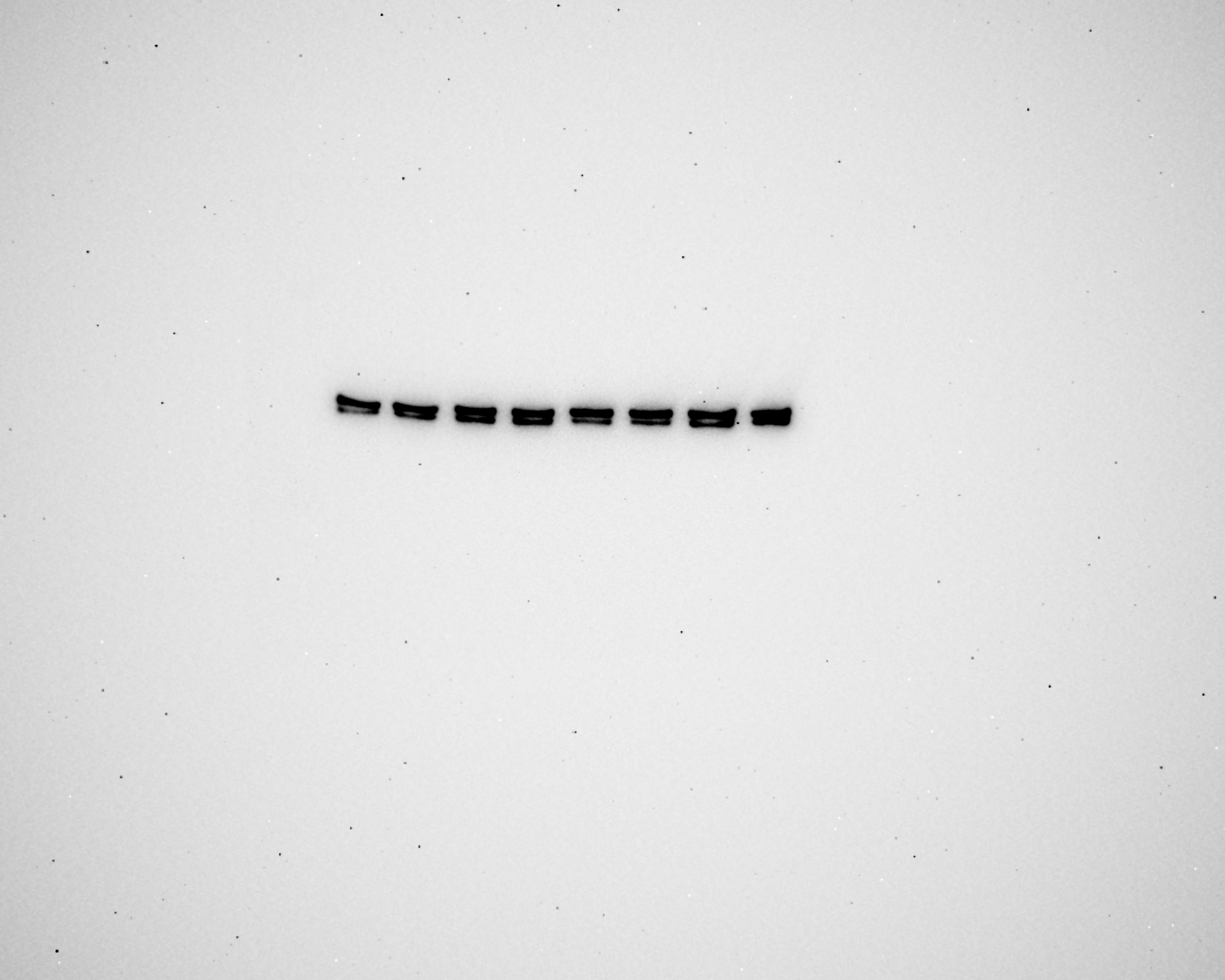

Supplement: Supplementary file 2 [file Image1.jpeg]

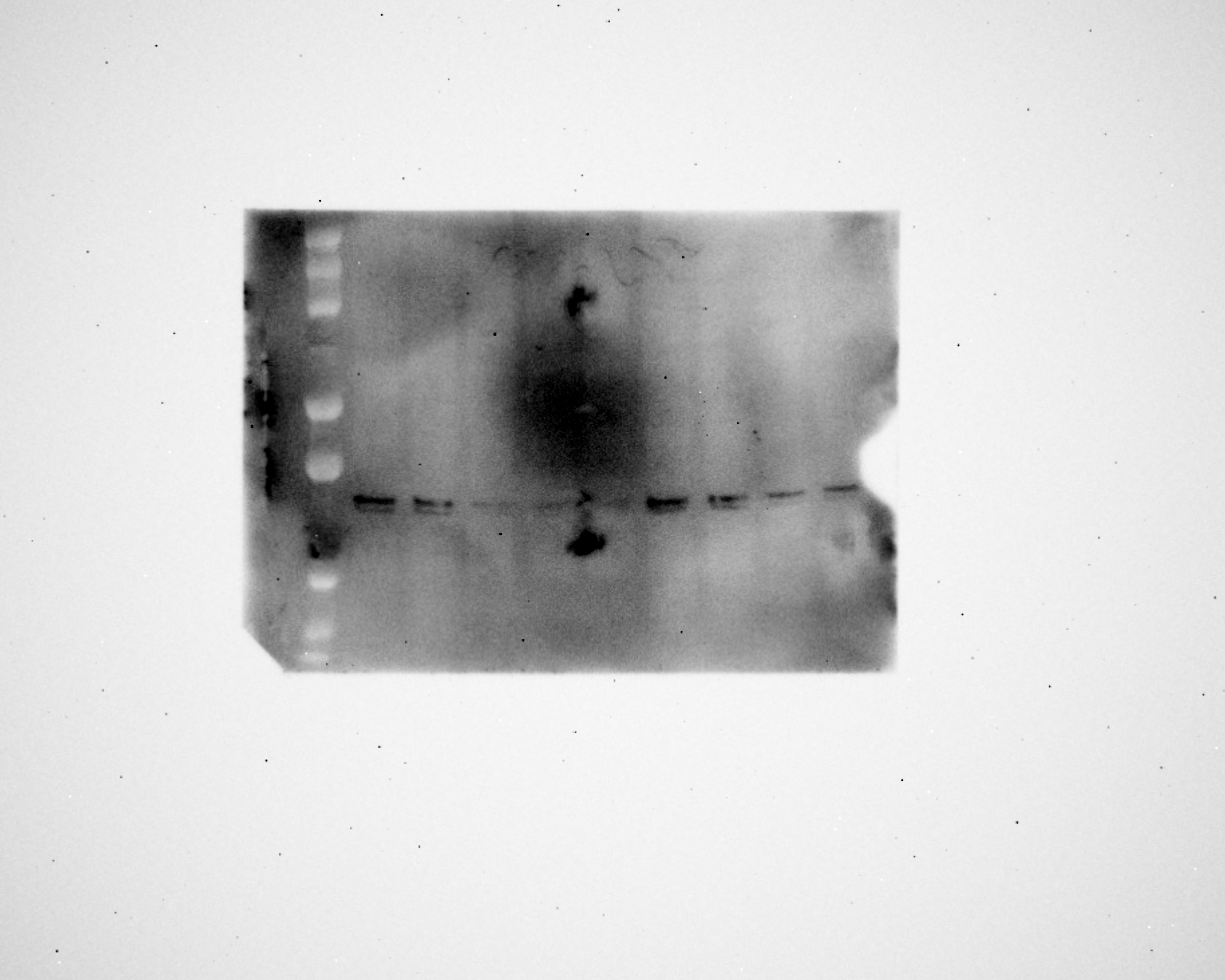

Supplement: Supplementary file 3 [file Image4.jpeg]

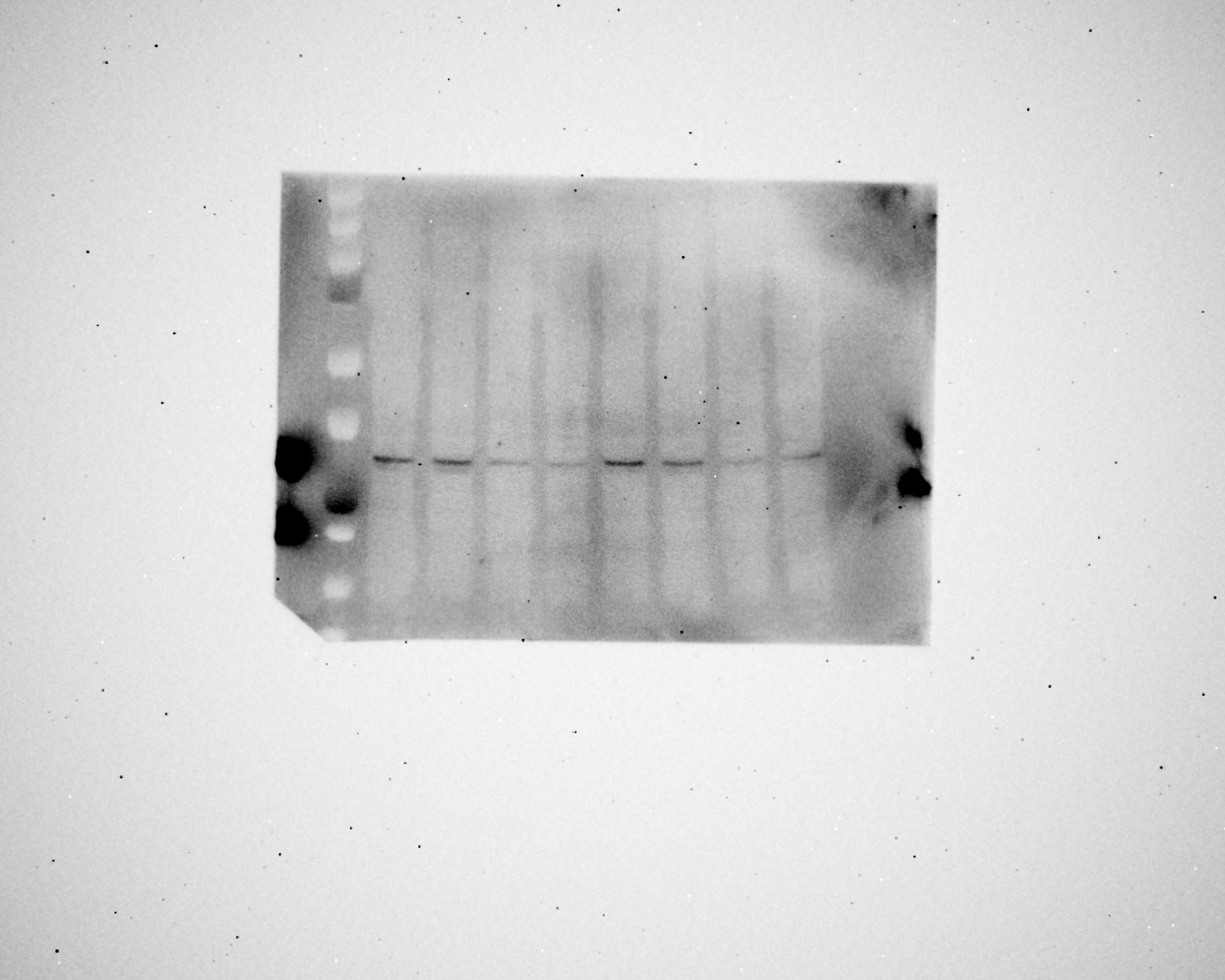

Supplement: Supplementary file 4 [file Image2.jpeg]

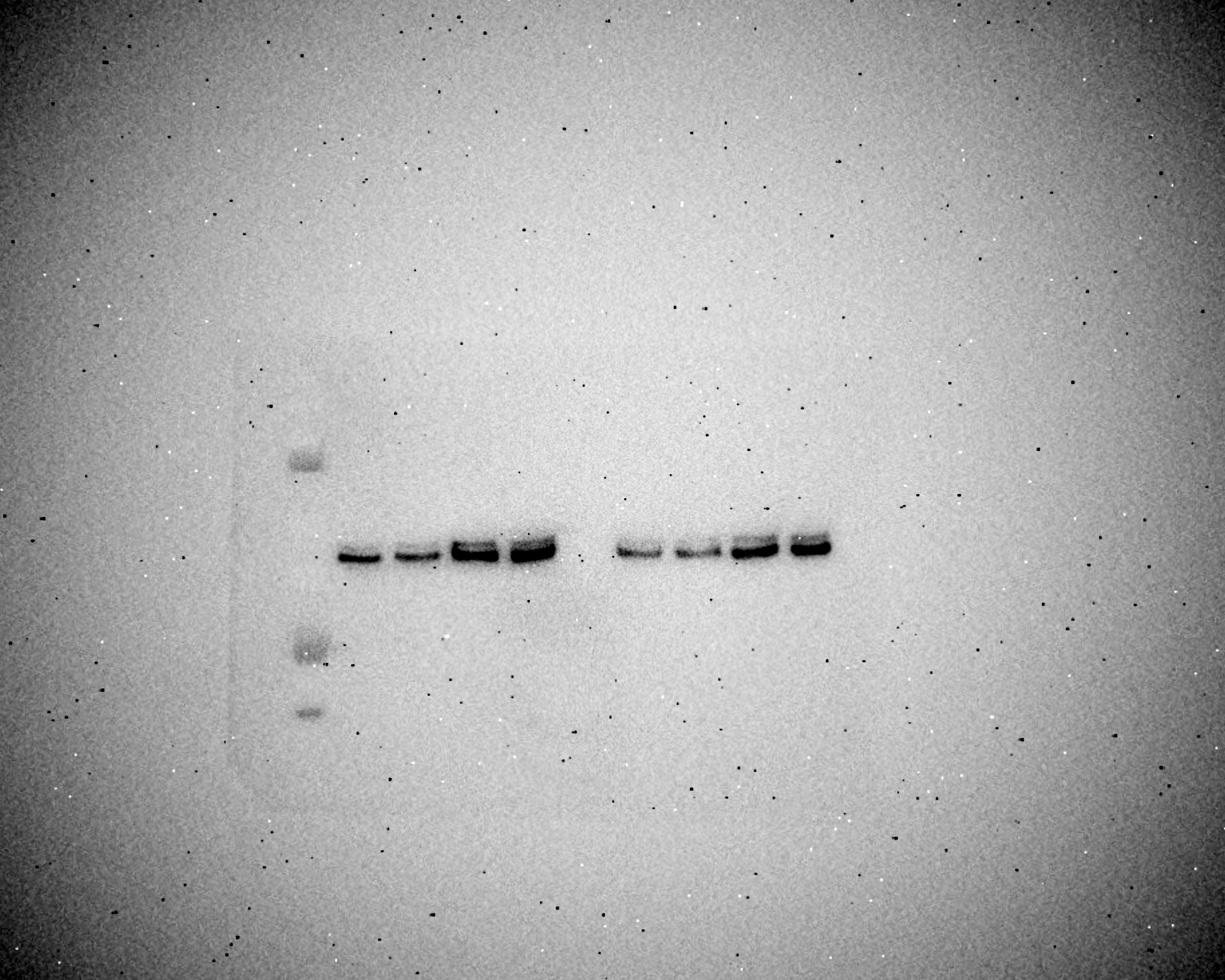

Supplement: Supplementary file 5 [file Image5.jpeg]
